# Non-ionizing measurement and quantification of bell-shaped chests in spinal muscular atrophy: a pilot study

**DOI:** 10.3389/fped.2024.1256445

**Published:** 2024-02-02

**Authors:** Israel Amirav, Neta Rabin, Sapir Levi, Ronly Har-Even Cohn, Yotam Lior, Shelly Shiran, Liora Sagi, Aviva Fatal, Alon Zvirin, Yaron Honen, Moran Lavie, Ron Kimmel

**Affiliations:** ^1^ Pediatric Pulmonology Unit, Dana-Dwek Children’s Hospital, Tel-Aviv Sourasky Medical Center, Tel Aviv, Israel; ^2^ Affiliated to the Faculty of Medicine, Tel Aviv University, Tel Aviv, Israel; ^3^ Division of Anesthesia, Intensive Care, and Pain Management, Tel-Aviv Sourasky Medical Center, Tel Aviv, Israel; ^4^ Radiology Department, Tel-Aviv Sourasky Medical Center, Tel Aviv, Israel; ^5^ Department of Computer Sciences, The Technion, Israel Institute of Technology, Haifa, Israel; ^6^ Department of Electrical and Computer Engineering, The Technion, Israel Institute of Technology, Haifa, Israel

**Keywords:** bell-shaped, chest, spinal muscular atrophy, chest geometry, depth camera

## Abstract

**Background:**

Spinal Muscular Atrophy (SMA) is manifested by deformation of the chest wall, including a bell-shaped chest. We determined the ability of a novel non-ionizing, non-volitional method to measure and quantify bell-shaped chests in SMA.

**Methods:**

A 3D depth camera and a chest x-ray (CXR) were used to capture chest images in 14 SMA patients and 28 controls. Both methods measure the distance between two points, but measurements performed by 3D analysis allow for the consideration of the curve of a surface (geodesic measurements), whereas the CXR allows solely for the determination of the shortest path between two points, with no regard for the surface (Euclidean measurements). The ratio of the upper to lower chest distances was quantified to distinguish chest shape in imaging by both the 3D depth camera and the CXR, and the ratios were compared between healthy and SMA patients.

**Results:**

The mean 3D Euclidean ratio of distances measured by 3D imaging was 1.00 in the control group and 0.92 in the SMA group (*p* = 0.01), the latter indicative of a bell-shaped chest. This result repeated itself in the ratio of geodesic measurements (0.99 vs. 0.89, respectively, *p* = 0.03).

**Conclusion:**

The herein-described novel, noninvasive 3D method for measuring the upper and lower chest distances was shown to distinguish the bell-shaped chest configuration in patients with SMA from the chests of controls. This method bears several advantages over CXR and may be readily applicable in clinical settings that manage children with SMA.

## Introduction

Spinal muscular atrophy (SMA) is an autosomal recessive neuromuscular disease characterized by degeneration of alpha motor neurons in the spinal cord, resulting in progressive proximal muscle weakness and paralysis. SMA is caused by a defect in the survival motor neuron (*SMN*) 1 gene. The level of functional SMN protein, which is reportedly related to the clinical severity of the disease, depends on the number of copies of the paralogous *SMN*2 gene (1). SMA is classified into clinical groups based on age at onset and maximum motor function that had been achieved: type 1 or Werdnig-Hoffmann disease (very weak infants unable to sit), type 2 (non-ambulant children able to sit unsupported), type 3 (ambulant children), and type 4 (adulthood onset) (2). SMA type 1 is the most common and most severe type, accounting for about 50% of all SMA patients. Classically, the onset of clinical signs among infants with SMA type 1 is before 6 months of age. These patients never acquire the ability to sit unsupported and generally do not survive beyond 2 years of life if no intervention is provided (3).

Pulmonary disease is the leading cause of morbidity and mortality in SMA type 1. Respiratory failure is mostly caused by very weak intercostal muscles, whereas the diaphragm is relatively spared. The external intercostal muscles, in particular, are considered accessory muscles, and they constitute a reserve system that is recruited when the expansion of the ribcage and/or respiratory effort needs to be increased (4). For these reasons, severe intercostal and abdominal muscle weakness, as characterized in SMA, leads to reduced ribcage expansion and inefficient cough in SMA (5). In the first years of life, the chest wall is very compliant. SMA is characterized by a lack of opposition of the intercostal muscles against the function of the diaphragm, and therefore the chest wall appears collapsed and bell-shaped (6). This results in poor airway clearance, alveolar hypoventilation, atelectasis, and decreased lung distensibility. Patients initially have recurrent respiratory infections, followed by nocturnal oxygen desaturation, nocturnal hypoventilation, and then daytime hypercarbia (7–9). Many patients with SMA type 1 eventually require long-term ventilatory support (8).

Pulmonary function testing (PFT) provides important information on respiratory physiology and helps guide clinical management of patients with SMA. It can also help assess the impact of emerging new treatments, such as gene therapy, on the patient's pulmonary disease status. The most commonly used forms of PFT, such as spirometry and body plethysmography require patient cooperation and therefore do not apply to very young children. These limitations emphasize the need for less demanding forms of evaluation that involve minimum patient cooperation (10, 11).

Deformations of the chest wall, including a bell-shaped chest are common in SMA. Evaluating bell-shaped chest wall deformity is of clinical significance since this may be one of the first signs of respiratory insufficiency and a need for consideration of initiation of noninvasive ventilation (NIV) in SMA patients (6). There have been some suggestions for an association between NIV use and reduced chest wall deformity and possibly improved lung development (12, 13).

There is sparse scientific data on the quantification of chest geometry in children, particularly those with SMA, and the available methods to assess it are limited. Chest radiography, for example, has been used for this purpose in children with spastic quadriplegic cerebral palsy (14), but it involves ionizing radiation. Opto-electronic plethysmography (OEP) has recently been suggested by LoMauro et al. as a noninvasive method to assess chest geometry in SMA. The authors demonstrated a correlation between a bell-shaped chest index and the presence of asynchronous breathing, and the degree of SMA severity (15). While the method used in that study was noninvasive, it required multiple points of measurement and the use of special equipment and devices, which are rarely available in the usual clinical setting.

Respiratory assessment, including awareness of an early developing bell-shaped chest in youngsters with SMA, is important and necessary to better understand the effects of treatments on respiratory function in these patients. A recent study demonstrated the importance of bell-shape assessment as an outcome measure in studying the efficacy of the new antisense oligonucleotide that modifies the splicing of the SMN2 mRNA transcript (Nusinersen) treatment (16, 17). We aimed to develop a new, non-ionizing, non-volitional method to measure and quantify the dimensions of bell-shaped chests in SMA. In the present pilot study, we tested the feasibility of a new, method of assessing bell-shaped chests in patients with SMA.

## Methods

### Patient population

In this prospective study, we recruited children with genetically and clinically confirmed SMA types 1 and 2 who received any of the new treatment options, such as splicing modulation of *SMN2* and *SMN1* gene replacement by gene therapy. These patients were recruited from our multidisciplinary SMA clinic at the Tel Aviv Medical Center (TAMC). All patients received care according to the consensus on standards of care in SMA (18). Study exclusion criteria included: Previous spinal surgery, acute respiratory distress, and permanent mechanical ventilation dependence. We also excluded patients confined to wheelchairs for whom lying on a bed for image acquisition could be uncomfortable. A control group of children (1:2) attending our general medicine outpatient clinic (for non-neuromuscular and non-respiratory issues) was also included. They were age-matched to the study group. Some of the control group children had CXRs for reasons unrelated to the current study (primarily as part of an anesthetic evaluation prior to non-respiratory surgical procedures).

Given that this was a pilot and feasibility study, it is common to conduct a smaller-scale investigation to assess the practicality and potential challenges of a larger study. In such cases, the focus is often on assessing whether the proposed methods are viable and feasible. Furthermore, as the target population in this case was rare or challenging to recruit, we did not have the ability to achieve a larger sample size within a reasonable timeframe. Thus, we have proceeded with the available participants, bearing in mind the potential for a larger sample size in the future.

The study received approval from the TAMC ethics committee (approval number 0348-18-TLV) and is registered at the Ministry of Health clinical trials registry (MOH_2019-03-14_006019). Informed consent was obtained from all participants or their parents. All image-capturing sessions were completed within the regularly scheduled clinic visit and required minimal additional time.

This study was done in collaboration with the geometric image processing lab at the Department of Computer Science of the “Technion,” Israel Institute of Technology (Haifa, Israel).

### 3D image acquisition

**Figure 1:**
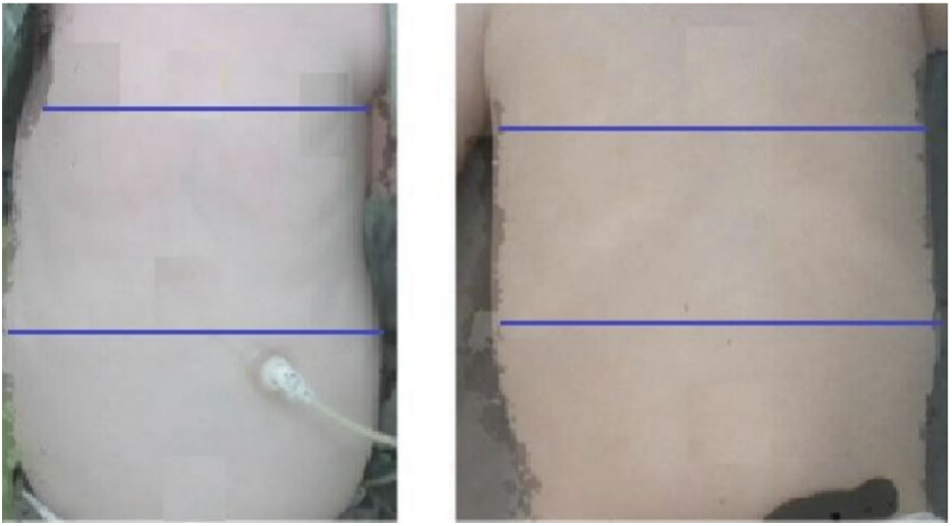
An SMA patient on the left and a healthy control on the right. These frames were taken by the Intel (RealSense™) 3D depth camera. The blue lines illustrate measurement distances. The levels of the lines were standardized using the nipples for the upper line and the umbilicus for the lower line. (Although these markers are not seen in the printed images, they were clearly identified during the RealSense analysis).

**Figure 2:**
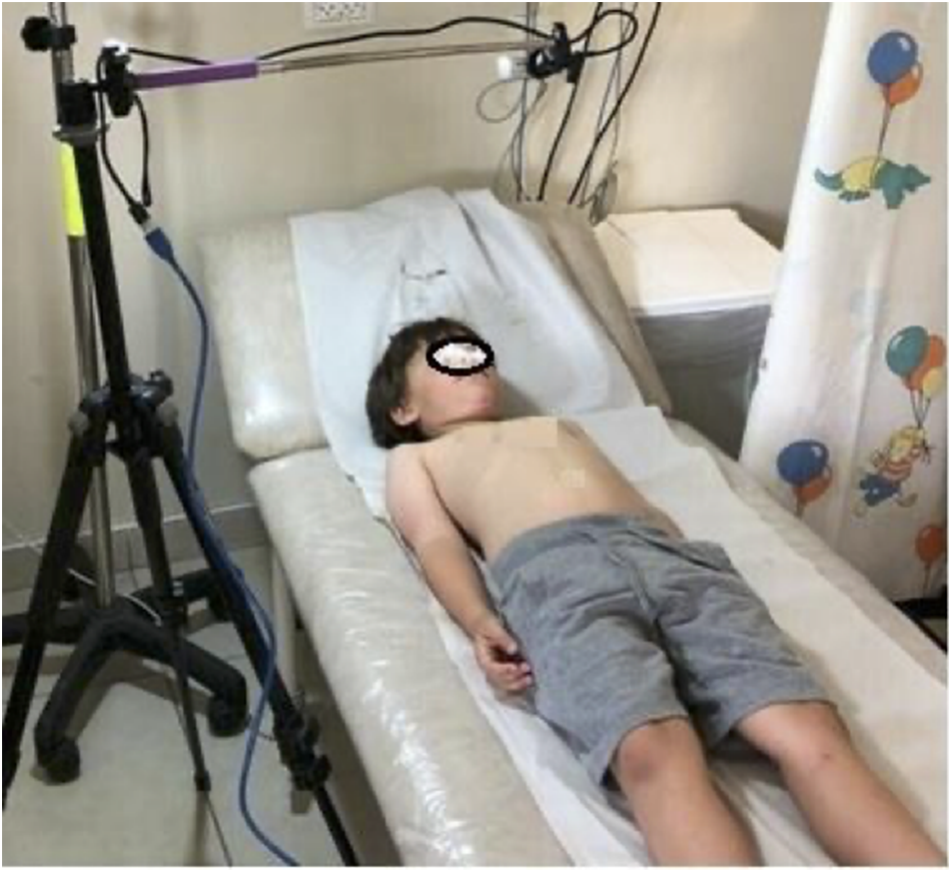
Intel (RealSense™) 3D depth camera posted on a tripod at beside.

We used an innovative three-dimensional (3D) depth camera (Intel RealSense 435™) to obtain photographs of upper and lower chests from which the shape of the chest (bell-shaped or normal) can be determined. The camera we used is now commercially available (at a cost of $150) and simple to use. It is based on RealSense technology, which is increasingly utilized in various clinical research studies (19), and we implemented it in our previous study on the design of aerosol face masks for children (20). The patients were asked to lie on a bed with the chest and abdomen facing up (Figure 1). The camera was posted on a tripod at the bedside (Figure 2) to record the patient's frontal chest and abdomen motions during 60 s of normal and quiet breathing. The camera can capture and stream the depth data of moving objects (including chest motions), thus providing high depth-perception accuracy during motion. The camera captured ∼15 frames per second and 480 × 640-pixel resolution, and the videos were reviewed frame-by-frame to ensure optimal imaging for further measurements and analyses. For each video recording, 5 consecutive frames characterized by smooth, quiet breathing were selected for analysis.

### Chest shape determination

Assessment of the chest shape was based on the ratio between two horizontal distances: one distance measured between two points on the upper chest, and the second distance measured between two points on the lower chest. The upper horizontal distance was calculated between the right and left two anterior axillary lines at the nipple height, equivalent to the right and left 4th ribs. The lower horizontal distance was calculated between the chest contour edges at sub-xiphoid height, equivalent to the right and left 8th costal ribs. Chest distance ratios smaller than 1 indicated a bell shape, and a ratio larger or close to 1 indicated a rectangular shape (Figure 1).

### Analysis

**Figure 3:**
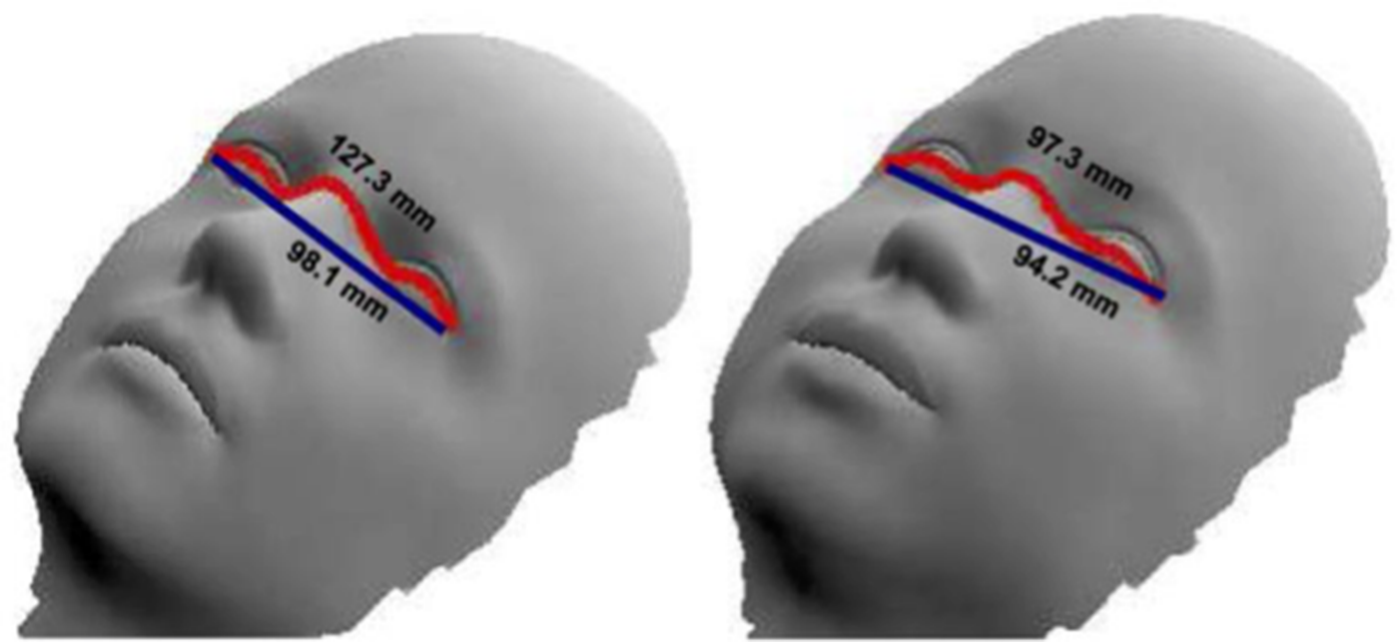
An example demonstrating how we measure Euclidean and geodesic distances on a hypothetical face. Euclidean distance defined by the line in dark blue vs. geodesic distance defined by the length of the path in red. [with permission from (21)].

The geometric image processing lab of the Technion developed an application to measure Euclidean and geodesic distances between two selected points on a selected image (frame). Euclidean distance is the ordinary straight-line distance between two points, whereas geodesic distance is defined by the length of the shortest path between two points on a surface. While the Euclidean distance completely ignores the shape or curve of the surface when finding a path from the starting point to the endpoint, the path is constrained within the given shape for the geodesic distance. An example showcasing the difference between the Euclidean and geodesic distances is included in Figure 3. As previously mentioned, we selected 5 consecutive frames characterized by smooth, quiet breathing and calculated an average Euclidean and geodesic distance for each video recording.

### Comparison of the 3D and CXR images

In order to compare the accuracy of the newly developed technique to assess bell shape deformity, we have also assessed the bell-shape deformity from Chest x-ray (CXR). CXRs of patients were obtained as part of routine clinical care of SMA patients and performed within 3 months of 3D image acquisition. CXRs for the controls were obtained for clinical reasons unrelated to the study. All CXRs were obtained postero-anterior (PA) in the supine position with particular attention to body positioning.

**Figure 4:**
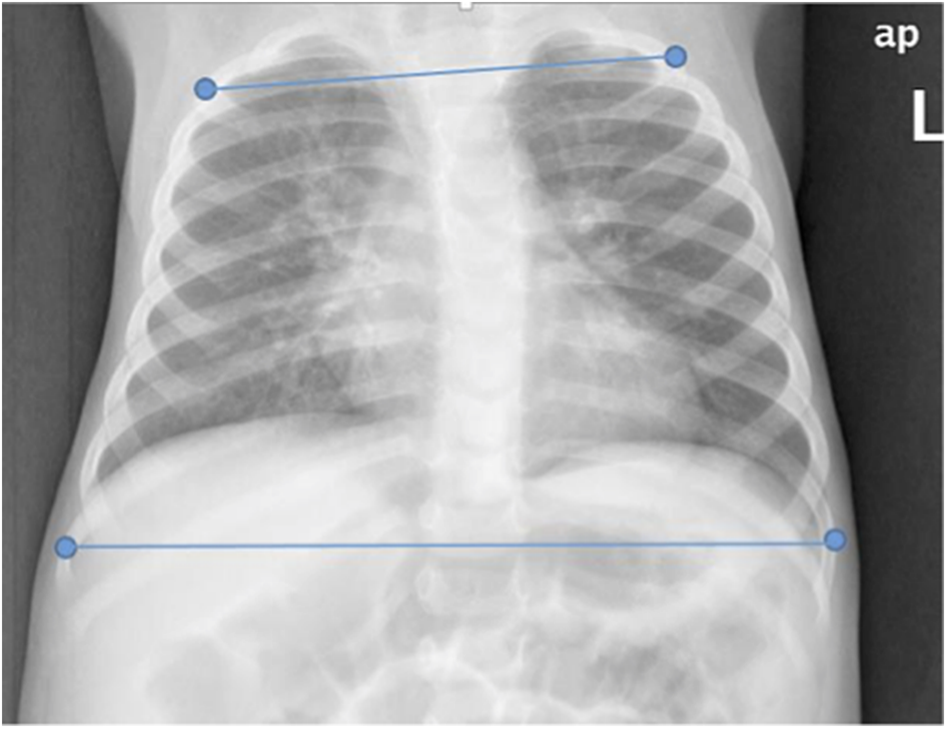
Chest x-ray with markings on the edges of the 2nd and 9th ribs for distance measurements. Similarly, we also measured distances at edges of the 4th and 8th ribs.

Horizontal chest distances (upper and lower chest) and their ratios were measured from CXRs for all study participants by a certified pediatric radiologist (SL). In contrast to the RealSense measures, which were obtained from points on the chest surface, distances on CXRs were measured on discrete points of reference located below the body's surface. These points consisted of the lateral edges of the 2nd and 9th ribs (Figure 4). To enrich the comparison, we also measured distances in the CXR of the lateral edges of the 4th and 8th ribs, as these points are more equivalent to the levels where the 3D measurements were taken. All distances were calculated on the anteroposterior view of CXRs taken with the subject in the supine position.

## Statistical analysis

The data collected in this study were consolidated using prevalence tables. Quantitative variables with normal distribution were presented as means with standard deviations. Arranged or quantitative variables without a normal distribution were presented as a median with inter-quarterly intervals. Categorical variables were presented as a count and percentage from the group. The preferred data analysis method for quantitative variables was parametric. However, the small sample size does not allow for parametric assumptions, whereupon the Mann–Whitney test was used to compare the data. As appropriate, categorical variables were assessed with Pearson's *χ*^2^ or the Fisher Exact Test. Correlation between variables was performed using the Pearson or Spearman correlation tests, as appropriate. All statistical tests were performed for *α* = 0.05 bilaterally unless otherwise stated. All *p*-values were rounded to two digits after the decimal point. Data analysis was performed using IBM SPSS software.

## Results

Fourteen SMA patients and 28 controls were recruited into the study. Table 1 displays their demographics.

**Table 1:** Cohort characteristics.

	Entire cohort	Healthy controls	SMA patients	*p*-value
	(*n *= 42)	(*n *= 28)	(*n *= 14)	
Mean age (years), (SD)	3.07 (3.05)	2.88 (2.97)	3.44 (3.3)	0.44
Mean weight (kg), (SD)	14.8 (12.02)	17.5 (15.48)	11.71 (5.15)	0.33
Mean height (m), (SD)	1 (0.34)	1.15 (0.39)	0.88 (0.24)	0.12
Mean BMI (SD)	17.15 (5.98)	19.65 (7.89)	15.01 (2.34)	0.053
Sex, *n* (%)				
Female	17 (40.5%)	10 (35.7%)	7 (50%)	0.37
Male	27 (59.5%)	18 (64.3%)	7 (50%)	

SD, standard deviation.

There were no significant differences between the SMA and control groups in age (3.44 ± 3.3 vs. 2.88 ± 2.97 years, respectively, *p *= 0.44), weight (11.71 ± 5.15 vs. 17.5 ± 15.48 kg, *p* = 0.33), BMI (19.65 ± 7.89 vs. 15.01 ± 2.34, *p* = 0.053) nor sex (50% vs. 35.7% females, *p* = 0.37).

Eleven of the 14 SMA patients had SMA type 1 (78.6%), and 3 had SMA type 2 (21.4%). The SMA patients' clinical characteristics are displayed in Table 2.

**Table 2:** SMA cohort characteristics.

SMA type, *n* (%)	
1A	1 (7.1%)
1B	6 (42.9%)
1C	4 (28.6%)
2	3 (21.4%)
Noninvasive ventilation (BIPAP), *n* (%)	
None	5 (35.7%)
Nighttime only	2 (14.3%)
Over 12 h per day	7 (50%)
Nusinersen Tx, *n* (%)	10 (71.4%)
Mechanical insufflation–exsufflation (MI-E), *n* (%)	3 (71.4%)
CHOP-INTEND (Max 64), mean (SD)	45 (13)
Scoliosis	1 (7.1%)
Mean age of first nusinersen Tx (years), (SD)	2.65 (3.63)
Mean number of nusinersen Tx per patient, mean (SD)	8 (2.2)
Mean duration of nusinersen Txs per patient (years), (SD)	1.6 (0.9)
Onasemnogene aberparvovec, *n* (%)	4 (28.6%)
Mean age at 1st onasemnogene aberparvovec Tx (years), (SD)	1.15 (0.95)

Tx, treatment; SD, standard deviation; CHOP-INTEND, the Children's hospital of philadelphia infant test of neuromuscular disorders.

SMA type 1 patients were divided according to age at symptom onset: *1A* age at onset was younger than two weeks (1 patient), *1B* between two weeks and six months (6 patients), and *1C* older than six months (4 patients). Nine of fourteen patients (64.3%) were non-invasively ventilated, two only at nighttime, and the other seven partially during daytime. All patients received at least one of two newly approved medications for SMA (i.e., nusinersen and onasemnogene aberparvovec). Further clinical characteristics of these patients (ventilatory support, functional assessment, scoliosis, and treatments) are described in Table 2.

In the analysis of 3D imaging, the mean Euclidean ratio of distances was 1 in the healthy group and 0.92 in the SMA group (*p* = 0.01). This result repeated itself in the geodesic measurements (0.99 vs. 0.89, *p* = 0.03).

All 14 SMA children underwent a CXR analysis, but it was available for only 14 (50%) of the controls. Prudence dictated a reluctance to subject controls to superfluous diagnostic interventions. This consideration underscores our commitment to ethical research practices and minimization of undue exposure to investigative procedures among healthy individuals. The most recent (from the time of 3D image acquisition) CXRs of the SMA patients were selected for analysis. No geodesic analysis was performed on the CXRs, given their 2D nature. We measured the Euclidean distance ratios from CXR images in both research groups using the 2nd vs. the 9th ribs ratio and the 4th vs. the 8th ribs ratio. The mean Euclidean ratio of the 2nd vs. the 9th ribs was 0.6 in healthy patients and 0.6 in the SMA group (*p = *0.92)*.* This result was consistent with the Euclidean ratio of the 4th and 8th ribs (0.79 vs. 0.8, *p = *0.61)*.* Within the SMA group, both CXR ratios significantly differed from the 0.92 ratio found in the 3D Euclidean ratio: 0.6 for ribs 2–9 (*p*-value 0.001) and 0.8 for ribs 4–8 (*p*-value 0.004).

## Discussion

Pulmonary complications are the primary cause of morbidity and mortality in SMA patients (22), yet pulmonary function assessment is very limited in infants and young children with SMA. Assessing thoracic wall deformation such as bell-shape has been recently suggested to be a surrogate for the commonly used PFTs, due to the limited compliance of this population group (15). The results of our pilot study confirmed the feasibility of an innovative technique for the noninvasive and non-ionizing assessment of bell-shaped chest in young children with SMA.

As shown in Table 3, there were two variables analyzed in this study: the *actual* (absolute) sizes of the upper and lower chest horizontal dimensions (distances), and the *ratio* between these distances. Both types of measurements were obtained using two different technologies- CXR vs. 3D image analysis.

**Table 3:** Results of the 3D and CXR-based measurements and their ratios.

	Healthy controls	SMA patients	*p*-value
	(*n *= 28)	(*n *= 14)	
Mean EUC (cm), (SD)			
Upper Chest	15.83 (3.98)	14.47 (2.45)	0.14
Lower Chest	15.88 (3.72)	15.88 (3.13)	0.92
Ratio	1 (0.09)	0.92 (0.07)	**0** **.** **01**
Mean GEO (cm), (SD)			
Upper Chest	19.7 (4.84)	18.27 (3.05)	0.34
Lower Chest	20.07 (4.83))	20.8 (4.55)	0.66
Ratio	0.99 (0.13)	0.89 (0.1)	**0** **.** **03**
Mean x-ray distance (cm), (SD)			
2nd Rib	9.25 (2.45)	10.96 (1.94)	0.047
9th Rib	15.42 (3.32)	18.16 (2.53)	**0** **.** **02**
Ratio	0.6 (0.04)	0.6 (0.05)	0.92
Mean x-ray distance (cm), (SD)			
4th Rib	11.83 (3.43)	14.41 (2.57)	**0** **.** **03**
8th Rib	14.92 (3.45)	17.92 (2.61)	**0** **.** **02**
Ratio	0.79 (0.07)	0.8 (0.05)	0.61

EUC, euclidean; GEO, geodesic; cm, centimeter.

Bold values denote significant difference.

With regard to the absolute values of the upper/lower chest dimensions - it is worth noting that the CXR technique showed broader chest dimensions (both in the upper (2nd and 4th rib levels) as well in the lower chest (9th and 8th ribs) in the SMA patients (Table 3). Given the natural growth and development of the ribs with age, this observed difference may be related to the older age of the SMA group (23). Despite the differences in absolute values, the *ratios* between upper and lower chest dimensions (bell-shape indices) obtained from the CXR were similar between the two groups.

Park et al. previously tried to quantify the upper ribcage's reduced growth by measuring the ratio of the ribcage apex (upper chest) and the base (lower chest) diameters on anteroposterior views of a CXR (14). Their method was tested on children with severe spastic quadriplegic cerebral palsy (CP) without scoliosis and on healthy peers. The study's upper/lower diameter ratio was 0.62 for the patients compared to 0.66 for the healthy controls. These values are very close to those we obtained from the CXRs for the 2nd vs. 9th ribs ratio in our SMA patients and healthy controls (0.60 and 0.60, respectively).

Interestingly, unlike their study, we did not observe a significant difference in the CXR-obtained ratios between our SMA patients and controls.

Possible explanations for the inability to detect a significantly different CXR ratio in our study compared to the Park study may first relate to the different pathology of the patients included-SMA vs. CP, with CP having a more drastic effect on chest deformity. Another contributing factor may be the clinical condition of the patients. While the SMA patients in our study were treated starting in early childhood, the CP patients were not treated at all (14). Lastly, the difference in our results may be related to our much smaller sample size (14 patients and 28 controls vs. 112 patients and 112 healthy controls in the Park study) (14).

In contrast to the CXR technique, the 3D analysis did not reveal a difference in absolute dimensions but did reveal a significant difference in the bell shape index (ratio) between the groups.

All of our SMA children were diagnosed at an early stage in our tertiary medical center. They were followed up regularly and received optimal treatment with both ventilation and new compounds that have been shown to successfully restore SMN protein production in motor neurons, either by enhancing the production of the SMN protein or by direct replacement of the *SMN1* gene. Therefore, it was not surprising that the incidence of the canonical bell-shaped chest (as evident from CXR) was rare in our cohort, that its prevalence did not significantly differ from the controls, and that it could be detected only by more sensitive methodologies, such as the 3D imaging. Since CXR imagining relies solely on bone shadowing, it is well within reason to assume that imaging techniques which also account for soft tissues, as is the case for 3D imaging, would be sufficiently sensitive to detect muscle atrophy changes as well (24). Whereas the CXR measures the distance between the ribs, the 3D image analysis also takes into account the soft tissues. Connective tissue in the chest area solidifies in children with SMA, and therefore, the identification of a bell-shaped chest may require this extra parameter in image analysis.

CXR has a significant drawback in pediatric care since it requires repeated exposure to ionizing radiation. Despite the small sample size, the significant difference observed in our 3D imaging arm emphasizes the advantage of this method compared to CXR for detecting a developing bell-shaped chest.

LoMauro et al. used non-ionizing radiation optoelectronic plethysmography (OEP) to measure and quantify the bell-shaped index (ratio of upper to lower chest distances) in SMA children (15). While OEP technology is innovative and radiation-free, it is based on multiple infrared video cameras with 36 retro-reflective markers that need to be placed on the patient. Thus, it is neither readily available nor easy to use in daily clinical practice.

We set out to develop a new method for measuring the chest shape in SMA type 1 and 2 in a noninvasive, radiation-free, simple, and user- and patient-friendly manner by using an innovative 3D camera. Using our method, we found a significant difference between the chest shape of SMA types 1 and 2 compared to those of healthy controls. These findings suggest that the bell-shaped index, as determined in the present study, may be a good surrogate for the action of ribcage muscles in SMA. Given technological confirmation, with careful attention to standardization in patient positioning, lighting conditions, and image acquisition, clinically relevant 3D imaging is expected to be deployed in most smart mobile phones. Consequently, the diagnosis of the bell-shaped chest can become accessible to physicians in clinical settings, including in countries with scarce resources.

A few years ago, Ropass et al. suggested that the ratio of thoracic circumference (TC) to head circumference (HC) may be used as a clinical outcome for SMA patients (25). The authors reported that all those patients with TC/HC < 0.85 developed respiratory failure and died within 3 months. Although the technique used a very simple tape measure, it had some drawbacks compared to our study. Ropass et al. conducted their study with patients who were not supported by any ventilation mode, nor did they receive (at that time) any gene-modifying intervention (25). Furthermore, TC is just a single parameter and does not account for any chest deformities, including bell chest. Thus, the present study offers advancements over Ropass et al. by introducing a more sophisticated, objective and automated measurement technology, considering a broader range of patient characteristics, proposing a novel and validated (16) outcome measure (bell shape measurement), and providing a more comprehensive assessment of chest conditions in SMA patients.

The present method also has several advantages over the recently published OEP technology (14). It provides not only the ordinary straight-line distance between two points but also the geodesic distance, which considers the shape of the path between the starting point and the endpoint. And, as shown in our study, the geodesic distance is needed to diagnose a bell-shaped chest more accurately. A recent study showed the utility of geodesic measures obtained through the Real Sense technology in assessing Pectus excavatum in children (13). Moreover, these non-touch measures have/carry the potential to assess not only static dimensions but also dynamic, asynchronous breathing, another important respiratory parameter in SMA, which cannot be assessed using conventional devices (calipers or tape measures).

Some limitations should be acknowledged. The absence of a formal sample size calculation, the relatively small sample size, and the single-center nature of the study may limit the generalizability of our findings. Future studies with larger multi-centers and more diverse cohorts could provide more robust insights. Longer-term follow-up studies would be valuable to assess the stability or progression of the observed chest deformities.

Future research should also explore correlations between bell-shaped measurements and clinical parameters to provide a more comprehensive understanding of the clinical implications of bell-shaped chests.

In summary, conventional methods for quantifying and objectively assessing chest geometry often involve radiation exposure or necessitate the use of expensive devices. Our present study introduces the feasibility of a non-ionizing and non-volitional method for measuring and quantifying bell-shaped chests in SMA patients. This technology is not only readily available but also cost-effective and user-friendly, making it easily deployable in routine clinical practice.

The early acquisition of these measurements using feasible and simple noninvasive measures, holds the potential to significantly enhance decision-making in the monitoring of SMA patients.

## Data Availability

The raw data supporting the conclusions of this article will be made available by the authors, without undue reservation.
